# Menopausal Status Associated With Docetaxel-Induced Vascular Dysfunction in Breast Cancer Patients

**DOI:** 10.1016/j.jacc.2025.10.077

**Published:** 2026-02-17

**Authors:** Piotr Szczepaniak, Tomasz P. Mikolajczyk, Ewelina Jozefczuk, Diana Hodorowicz-Zaniewska, Joanna Streb, Jakub Jurczyk, Ryszard Nosalski, Mateusz Siedlinski, Paulina Sajdak, Karolina Brzuszkiewicz, Mateusz Gorski, Maciej Tomaszewski, Joanna Sulicka-Grodzicka, Iwona Laksa, Tomasz Grodzicki, Tomasz J. Guzik

**Affiliations:** a Department of Internal and Agricultural Medicine, Jagiellonian University Medical College, Cracow, Poland; b Breast Unit, Department of Surgery, Jagiellonian University Hospital, Cracow, Poland; c Department of Oncology, Jagiellonian University Medical College, Cracow, Poland; d Department of Toxicological Biochemistry, Jagiellonian University Medical College, Cracow, Poland; e Centre for Cardiovascular Science, Queen’s Medical Research Institute, University of Edinburgh, Edinburgh, United Kingdom; f Division of Cardiovascular Sciences, Faculty of Medicine, Biology and Health, University of Manchester, Manchester, United Kingdom; g Division of Medicine and Manchester Academic Health Science Centre, Manchester University NHS Foundation Trust Manchester, Manchester, United Kingdom; h Department of Rheumatology, Jagiellonian University Medical College, Cracow, Poland; i Department of Internal Medicine and Gerontology, Jagiellonian University Medical College, Cracow, Poland

**Keywords:** breast cancer, chemotherapy, docetaxel, endothelial dysfunction, menopause

## Abstract

**Background:**

Breast cancer chemotherapy increases cardiovascular (CV) risk, particularly in postmenopausal women. Although vascular damage, endothelial dysfunction, and oxidative stress are implicated, the role of menopausal status in vascular mechanisms of increased CV risk remains unknown. Accordingly, we investigated whether estrogens protect premenopausal women from neoadjuvant chemotherapy (TAC protocol)–induced endothelial dysfunction, focusing on the vascular effects of docetaxel. This is the first study to assess how menopausal status affects vascular responses to chemotherapy.

**Objectives:**

The main objective was to elucidate the pathophysiologic mechanisms by which TAC precipitates vascular dysfunction, with an emphasis on the influence of menopausal status.

**Methods:**

Vascular function, oxidative stress, and molecular pathways were evaluated in arterial segments from cancer-free breast tissue of premenopausal and postmenopausal women undergoing surgery with or without prior TAC. Complementary mechanistic studies were conducted in ovariectomized and control female C57BL/6J mice treated with docetaxel or placebo.

**Results:**

TAC-induced endothelial dysfunction, marked by reduced nitric oxide bioavailability, was observed in vessels from postmenopausal women, whereas premenopausal women were protected. The vessels of premenopausal women also resisted TAC-induced oxidative stress, showing significantly lower superoxide and hydrogen peroxide production as well as NOX4 NADPH-oxidase expression compared with their postmenopausal counterparts. No differences were noted between premenopausal and postmenopausal women in the non-TAC groups. At the molecular level, TAC resulted in lower inhibitory phosphorylation of endothelial nitric oxide synthase (eNOS) at threonine-495 and reduced rho-kinase activity in premenopausal women. In mice, docetaxel caused endothelial dysfunction, molecular changes, and hypertension only in ovariectomized animals, with no such effects in age-matched nonovariectomized controls.

**Conclusions:**

TAC-induced vascular dysfunction seen in breast cancer survivors is absent in premenopausal women, likely owing to estrogen-mediated protection against oxidative stress and eNOS inhibition.

Chemotherapy has transformed breast cancer prognosis: 10-year disease-specific survival now exceeds 80% in many countries.^1^ Yet survivors, particularly those treated with taxanes, anthracyclines, or human epidermal growth factor receptor 2 (HER2)–targeted agents, experience an excess burden of cardiovascular disease (CVD), which accounts for roughly one-third of noncancer deaths in these patients.^2^, ^3^, ^4^, ^5^ Although cardiomyocyte toxicity is well documented, far less is known about how modern chemotherapy perturbs the vascular endothelium,^6^ a key determinant of long-term cardiovascular risk. Docetaxel, a taxane widely used in neoadjuvant and adjuvant regimens, such as the breast cancer chemotherapy TAC (cyclophosphamide-docetaxel-doxorubicin), has emerged as a clinically relevant vascular toxin.^7^ Recent work from our group^7^ showed that in postmenopausal women, docetaxel stimulates endothelial production of reactive oxygen species (ROS) via up-regulation of NADPH oxidase isoforms NOX2 and NOX4. The resulting oxidative stress leads to rho-associated kinase (ROCK) activation and phosphorylation of endothelial nitric oxide synthase (eNOS) at threonine-495 (Thr495) and consequent endothelial dysfunction.^7^ In this context, docetaxel produced more pronounced vascular effects than doxorubicin or cyclophosphamide when each was tested individually ex vivo.^7^ Both anthracyclines and cyclophosphamide can increase oxidative stress, but our data identify docetaxel as the primary driver of vascular dysfunction in neoadjuvant TAC chemotherapy for breast cancer.^7^

Menopause represents an age-dependent phenotype and a key inflection point in cardiovascular risk.^8^^,^^9^ Estradiol enhances nitric oxide (NO) bioavailability, supports endothelial repair and vascular endothelial growth factor expression,^10^^,^^11^ and suppresses angiotensin II and endothelin 1 signaling as well as oxidative stress.^12^ Accordingly, its loss accelerates endothelial dysfunction and eliminates the female advantage in CVD that is observed before menopause.^8^^,^^9^ This manifests clinically in the impairment of flow-mediated dilation of peripheral arteries observed after menopause.^13^^,^^14^ Because aging is closely tied to estrogen loss, separating age-related vascular changes from estrogen-mediated effects is challenging because many vascular aging processes are hormone-dependent in both women and men. Importantly, the decline in endothelial function across the menopausal transition occurs independently from chronologic age: In a cohort of healthy women aged 50 to 59 years, flow-mediated dilation was markedly lower in late perimenopausal and early postmenopausal stages compared with early-perimenopausal women of the same age, whereas chronologic age explained only a small fraction of the variability in vascular function.^14^ Therefore, in the postmenopausal setting, vascular challenge by chemotherapy may have particularly damaging effects, translating into additional increases in vascular risk in cancer survivors.^2^, ^3^, ^4^^,^^15^ Although the incidence of breast cancer is higher in postmenopausal women,^16^ a rising proportion of breast cancer diagnoses occurs in premenopausal women, who often present with biologically aggressive disease and therefore receive intensive chemotherapy, including taxanes.^17^ Whether the premenopausal hormonal milieu protects against, or merely delays, chemotherapy-induced endothelial injury is unknown, a critical gap given the decades of life expectancy at stake.

We addressed that gap by interrogating human arteries harvested at mastectomy from premenopausal and postmenopausal patients treated with neoadjuvant docetaxel-containing chemotherapy, complemented by mechanistic studies in female murine models. We tested the hypothesis that menopausal status modifies docetaxel-induced NADPH oxidase activation and downstream eNOS dysregulation and linked those effects to ovarian hormonal status through mechanistic studies. Elucidating these interactions is essential for rational cardio-oncology risk stratification and for designing targeted vascular-protective interventions in young breast cancer survivors. We focused on docetaxel owing to its predominant vascular effects as previously described.^7^

## Methods

### Patients

**Figure 1:**
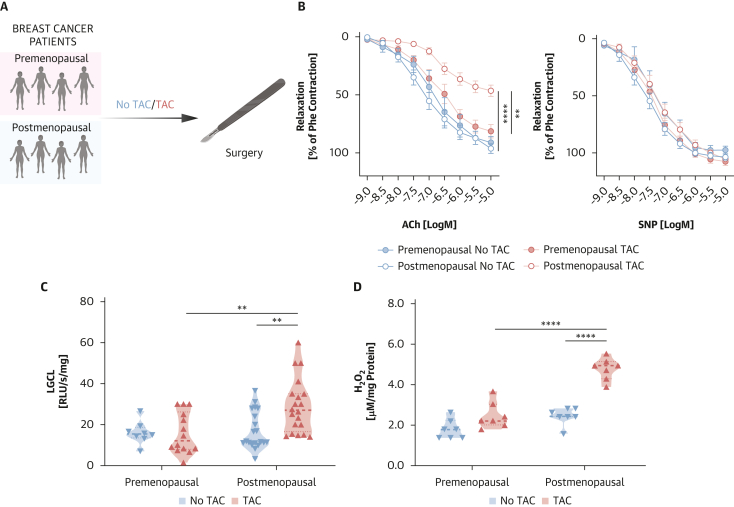
Effects of Menopausal Status on Neoadjuvant Chemotherapy–Induced Endothelial Dysfunction and Reactive Oxygen Species Production (A) Study design of human arteries collection in patients with breast cancer before and after menopause without neoadjuvant chemotherapy (No TAC) or after neoadjuvant chemotherapy (TAC) before surgery. (B) Average endothelium-dependent vasorelaxations curves to acetylcholine (ACh; 1 nmol/L to 10 μmol/L; left) and endothelium-independent relaxations to sodium nitroprusside (SNP; 1 nmol/L to 10 μmol/L; right) in blood vessels from premenopausal and postmenopausal women without TAC and who underwent TAC (n = 8-20). Data are expressed as mean ± SEM. ∗∗∗∗*P* < 0.0001 vs Postmenopausal No TAC; ∗∗*P* < 0.01 vs Premenopausal TAC. Data were analyzed by means of 2-way repeated-measures ANOVA with Sidak test. (C) Superoxide production measured with the use of lucigenin-dependent chemiluminescence (LGCL; n = 8-20/group) and (D) hydrogen peroxide (H_2_O_2_) production according to Amplex Red (n = 7/group) in arteries from patients before and after menopause without TAC or after TAC before surgery. ∗∗*P* < 0.01 vs Postmenopausal No TAC, Premenopausal TAC; ∗∗∗∗*P* < 0.0001 vs Postmenopausal No TAC, Premenopausal TAC; 2-way ANOVA with Tukey test. Data shown as mean ± SEM. Overall *P* values for repeated-measures 2-way ANOVA (B) for ACh: *P*_response_ < 0.0001, *P*_group_ < 0.0001, *P*_response×group_ < 0.0001; for SNP: *P*_response_ < 0.0001, *P*_group_ = 0.3596, *P*_response×group_ = 0.7034; for 2-way ANOVA (C) *P*_menopause_ = 0.0155, *P*_TAC_ = 0.0472, *P*_menopause×TAC_ = 0.0372; (D) *P*_menopause_ < 0.0001, *P*_TAC_ < 0.0001, *P*_menopause×TAC_ = 0.0005.

A total of 62 women diagnosed with locally advanced breast cancer were recruited before surgical treatment at the Department of General and Oncologic Surgery, University Hospital in Krakow. Both premenopausal (n = 22) and postmenopausal (defined as ≥12 months since last menstruation; n = 40) women were enrolled. The study cohort included patients undergoing surgery alone as well as those who received neoadjuvant chemotherapy using the TAC protocol followed by surgery (Figure 1A). The standard TAC regimen consisted of docetaxel (75 mg/m^2^ body surface area [BSA]), doxorubicin (50 mg/m^2^ BSA), and cyclophosphamide (500 mg/m^2^ BSA) administered intravenously every 3 weeks for an average of 8 cycles, with the last dose approximately 4 weeks before surgery. Inclusion and exclusion criteria followed the modified protocol of our recent study.^7^ Eligible participants were premenopausal or postmenopausal women with histologically confirmed locally advanced breast cancer without distant metastases, who were qualified for surgical treatment with or without TAC neoadjuvant chemotherapy and provided written informed consent. Patient selection was independent from tumor hormonal receptors or HER2 status. Women were excluded if they had a history of symptomatic cardiovascular disease (including atherosclerosis, coronary artery disease, heart failure, or valvular disease), chronic inflammatory or autoimmune conditions (such as asthma, atopic dermatitis, or rheumatoid arthritis), or any acute inflammatory illness within the preceding month. Additional exclusion criteria included a diagnosis of other malignancies, previous chemotherapy, hormone or radiotherapy before surgery, active alcohol or drug abuse, or lack of informed consent. The study protocol was approved by the Local Bioethics Committee of the Jagiellonian University (approval nos.: KBET/84/B/2009, 1072.6120.340.2020, and 1072.6120.52.2024).

### Human artery preparation

As previously described,^7^ arterial samples were collected from healthy mammary tissue during mastectomy. Arteries representing thoracic artery branches were isolated, immediately immersed in ice-cold Krebs-Hepes buffer, and carefully dissected under a microscope at 4 °C. Each vessel was processed individually, and *n* refers to the number of individual patients contributing samples.

### In vivo mouse experiments

Animal experiments adhered to ARRIVE guidelines and were approved by the Local Ethical Committee for Animal Experiments in Krakow (approval nos. 20/2016 and 529/2021). Female and male C57BL/6J wild-type mice (3.5-4.5 months old) were obtained from the Jackson Laboratory. Bilateral ovariectomy was performed in females according to standard protocols.^18^^,^^19^ Mice received intraperitoneal injections of docetaxel (10 mg/kg; Cayman Chemicals) or vehicle every 5 days for 3 weeks.^7^ Blood pressure was recorded with the use of the Visitech BP-2000 tail-cuff system on a heated (37 °C) platform. Animals were pretrained with 10 practice measurements, followed by 15 recordings per session every 2 days by an investigator blinded to group assignment.^20^ After the treatment period, animals were killed via CO_2_ inhalation, and perfused with cold phosphate-buffered saline solution. Aortic tissues were harvested for vascular function assays, ROS measurement, gene/protein analyses, and blood samples for 17β-estradiol quantification.

### In vitro endothelial cell culture

Human microvascular endothelial cells (ATCC CRL-3243) were cultured in Medium MCDB131 (Thermo Fisher Scientific) supplemented with 10 ng/mL epidermal growth factor (Thermo Fisher Scientific), 1 μg/mL hydrocortisone (Merck), 10 mmol/L glutamine (Thermo Fisher Scientific), and 10% Fetal bovine serum (Thermo Fisher Scientific) with the addition of a 1% mixture of penicillin and streptomycin (Thermo Fisher Scientific). After an initial 4 hours of withdrawal of growth supplements from the culture, cells were incubated with 100 nmol/L docetaxel or 100 nmol/L docetaxel and 100 nmol/L 17β-estradiol (Merck) or vehicle (solvent) for 24 hours at 37 °C in a humidified atmosphere with 5% CO_2_.

### Vascular function assays

Isolated 2-mm rings of human arteries or mouse aortas were mounted on a wire myograph (Danish Myo Technology models 750TOBS or 610M) according to previously published protocols.^7^^,^^21^ Vessels were preconstricted with 120 mmol/L KCl, followed by phenylephrine (to 60% to 70% of maximal contraction). Endothelium-dependent relaxation was assessed with increasing concentrations of acetylcholine (ACh; 1 nmol/L–10 μmol/L), and endothelium-independent responses with sodium nitroprusside (SNP; 1 nmol/L–10 μmol/L; Sigma-Aldrich).

### Lucigenin-enhanced chemiluminescence

Superoxide production was assessed with the use of lucigenin (5 μmol/L; Sigma-Aldrich) in Krebs-Hepes buffer.^7^^,^^20^ Human arteries were aerated (95% O_2_/5% CO_2_) for 20 minutes before measurement using a FB12 chemiluminometer (Berthold). Data were normalized to vessel dry weight and expressed as RLU/s per mg.

### Amplex Red assay

Vascular hydrogen peroxide (H_2_O_2_) levels were measured with the use of Amplex Red (100 μmol/L) and horseradish peroxidase (0.2 U/mL for human tissue, 0.1 U/mL for mouse aortas). Fluorescence (Ex: 560 nm; Em: 590 nm) or absorbance (560 nm) was recorded. H_2_O_2_ levels were normalized to protein content or dry vessel weight.^7^

### Western blotting

Human and mouse vascular fragments were homogenized in T-PER buffer with protease/phosphatase inhibitors (Thermo Fisher). Protein concentrations were determined by bicinchoninic acid assay (Thermo Fisher). Samples were separated by sodium dodecyl sulfate–polyacrylamide gel electrophoresis (Bolt 4%-12% Bis-Tris Plus gels), transferred to nitrocellulose membranes, and blocked in 5% skim milk or 3% bovine serum albumin. Membranes were incubated overnight at 4 °C with primary antibodies (eg, anti-eNOS, anti–phospho-eNOS [Thr495], anti-NOX4, anti–β-actin), followed by fluorescent secondary antibodies (IRDye 680LT/800CW; LI-COR). Imaging was performed with the Odyssey Fc Imager (LI-COR).

### Immunofluorescence

Frozen human arteries embedded in optimal cutting temperature compound were cryosectioned (8 μm) and mounted on glass slides. Sections were blocked (Protein Block and 0.5% Triton X-100), incubated overnight with anti-NOX4 (Novus), and stained with Alexa Fluor 594 secondary antibodies. Nuclei were visualized with the use of 6-diamino-2-phenylindole (Life Technologies).

### Rho-kinase activity

Rho-associated kinase activity in human artery lysates was quantified using the Rho Kinase Activity Assay Kit (Merck Millipore), normalized to protein concentration.

### Quantitative real-time polymerase chain reaction

Mouse aortas were preserved in RNAlater (Ambion), and RNA was extracted with the use of the Direct-zol MiniPrep Kit (Zymo Research) with DNase I treatment. Complementary DNA synthesis was performed using the High-Capacity cDNA Reverse Transcription Kit (Applied Biosystems). Quantitative real-time polymerase chain reaction (qRT-PCR) was conducted with TaqMan Gene Expression Master Mix and Assays on the 7900HT platform (Applied Biosystems), analyzed with RQ Manager 1.2.1 software, and normalized to housekeeping genes.

### ELISA

Plasma 17β-estradiol concentrations were measured with the use of a commercial ELISA kit (Enzo Life Sciences), according to the manufacturer’s protocol. Plasma was isolated from heparinized blood and stored at −80 °C until analysis.

### Statistical analysis

Data were analyzed with the use of Statistica 14.0.0 (TIBCO) and GraphPad Prism 10. Categoric data were compared by means of chi-square tests. Comparisons among multiple groups were conducted by means of 1-way or 2-way analysis of variance with Sidak or Tukey post hoc corrections. Results are reported as mean ± SEM, with statistical significance defined as *P* < 0.05.

## Results

### Menopausal status and chemotherapy-induced endothelial dysfunction and ROS production

To investigate the differences in TAC-induced endothelial dysfunction and oxidative stress between premenopausal and postmenopausal women, we analyzed arterial segments obtained from patients divided into 4 groups: premenopausal without TAC, premenopausal with TAC, postmenopausal without TAC, and postmenopausal with TAC. The groups were matched for major demographic and cardiovascular risk factors, as well as for tumor characteristics (with the exception of age and tumor size >5 cm; related to TAC criteria as well as double receptor positivity), and there were no significant differences in pharmacotherapy, hormonal treatment, or radiotherapy exposure (Table 1). Importantly, receptor positivity was not associated with differences in baseline endothelial function (Supplemental Figures 1 and 2). To avoid potential direct TAC toxicity and focus on chronic effects, arteries were collected approximately 1 month after the final TAC cycle (Figure 1A). Vascular reactivity was assessed with isometric tension studies to evaluate endothelium-dependent vasodilation (ACh) and endothelium-independent vasodilation (SNP). Consistently with our earlier findings,^7^ we confirmed that TAC significantly impaired ACh-mediated endothelial relaxation in postmenopausal patients. Importantly, arteries from premenopausal women treated with TAC did not exhibit endothelial dysfunction, indicating a protective effect of the premenopausal status (Figure 1B). Endothelial function was also preserved in all patients who did not receive TAC, regardless of menopausal status. Furthermore, endothelium-independent responses to SNP were unchanged across all groups, confirming that smooth muscle function was intact (Figure 1B). In a 2-way analysis of variance, the difference in ACh-induced dose-dependent relaxations between premenopausal and postmenopausal women treated with TAC remained significant after adjusting for age (Supplemental Table 1), although the close collinearity between age and menopause makes this distinction difficult to disentangle. An additional sensitivity analysis excluding patients receiving cardiovascular therapies, which showed borderline differences in prevalence (Table 1), yielded results consistent with those observed in the overall cohort (Supplemental Figure 3).

**Table 1:** Characteristics of Patients Before and After Menopause With Prior TAC and Without Prior TAC

	Premenopausal, No TAC (n = 8)	Premenopausal, TAC (n = 14)	Postmenopausal, No TAC (n = 20)	Postmenopausal, TAC (n = 20)	*P* Value
Age, y^a^	43.7 ± 6.1	40.0 ± 5.1	61.4 ± 7.2	58.0 ± 5.8	<0.001
SBP, mm Hg^a^	121.6 ± 9.6	119.4 ± 13.6	125.0 ± 11.4	125.0 ± 6.7	0.366
DBP, mm Hg^a^	79.5 ± 5.7	75.5 ± 6.5	76.9 ± 7.9	77.8 ± 7.0	0.605
HR, beats/min^a^	70.1 ± 5.7	71.3 ± 5.0	72.5 ± 10.3	73.0 ± 7.4	0.801
BMI, kg/m^2^^a^	24.4 ± 6.7	24.9 ± 4.5	25.7 ± 4.2	26.2 ± 3.4	0.720
Obesity^b^					0.567
Not overweight, BMI <24.9 kg/m^2^	6 (75.0)	9 (64.3)	11 (55.0)	10 (50.0)	
Overweight, BMI 25.0-29.9 kg/m^2^	1 (12.5)	5 (35.7)	8 (40.0)	7 (35.0)	
Obese, BMI >30 kg/m^2^	1 (12.5)	0 (0.0)	1 (5.0)	3 (15.0)	
Hypertension^b^	3 (37.5)	3 (21.4)	5 (25.0)	8 (40.0)	0.602
Diabetes mellitus^b^	0 (0.0)	0 (0.0)	2 (10.0)	3 (15.0)	0.344
Smoking^b^	1 (12.5)	1 (14.3)	4 (20.0)	4 (20.0)	0.939
Hypercholesterolemia^b^	1 (12.5)	0 (0.0)	5 (25.0)	2 (10.0)	0.185
Previous hormonal therapy^b^	0 (0.0)	0 (0.0)	0 (0.0)	0 (0.0)	
Previous radiotherapy^b^	0 (0.0)	0 (0.0)	0 (0.0)	0 (0.0)	
Stage^b^					0.024
0	0 (0.0)	0 (0.0)	0 (0.0)	0 (0.0)	
1	4 (50.0)	4 (28.6)	9 (45.0)	1 (5.0)	
2	3 (37.5)	4 (28.6)	5 (25.0)	4 (20.0)	
3	1 (12.5)	6 (42.8)	6 (30.0)	15 (75.0)	
Tumor size >5 cm^b^	0 (0.0)	3 (21.4)	1 (5.0)	7 (35.0)	0.043
ER^+^/PgR^+^^b^	8 (100)	11 (78.6)	17 (85.0)	10 (50.0)	0.017
HER2^+^^b^	1 (12.5)	6 (42.8)	4 (20.0)	8 (40.0)	0.250
ACEI/ARB^b^	0 (0.0)	0 (0.0)	3 (15.0)	5 (25.0)	0.114
Statins^b^	0 (0.0)	0 (0.0)	4 (20.0)	2 (10.0)	0.187
Alpha-blocker^b^	1 (12.5)	2 (14.3)	0 (0.0)	0 (0.0)	0.123
Beta-blocker^b^	0 (0.0)	0 (0.0)	4 (20.0)	6 (30.0)	0.063
Calcium channel blocker^b^	0 (0.0)	3 (21.4)	0 (0.0)	1 (5.0)	0.066
Nitrates^b^	0 (0.0)	0 (0.0)	0 (0.0)	1 (5.0)	0.545
Diuretics^b^	0 (0.0)	2 (14.3)	2 (10.0)	3 (15.0)	0.693
Opioids^b^	0 (0.0)	0 (0.0)	0 (0.0)	0 (0.0)	
Insulin^b^	0 (0.0)	0 (0.0)	0 (0.0)	1 (5.0)	0.545
NSAIDs^b^	0 (0.0)	0 (0.0)	0 (0.0)	0 (0.0)	
Antidiabetes medication^b^	0 (0.0)	0 (0.0)	2 (10.0)	3 (15.0)	0.344

Values are mean ± SD or n (%).

ACEI/ARB = angiotensin-converting enzyme inhibitor/angiotensin receptor blocker; BMI = body mass index; DBP = diastolic blood pressure; ER = estrogen receptor; HER2 = human epidermal growth factor receptor 2; HR = heart rate; NSAIDs = nonsteroidal antiinflammatory drugs; PgR = progesterone receptor; SBP = systolic blood pressure; TAC = neoadjuvant chemotherapy (cyclophosphamide-docetaxeldoxorubicin).

a Comparison between 4 groups by means of 1-way ANOVA.

b Comparison between 4 groups by means of chi-square test.

To explore the underlying mechanisms, we assessed vascular oxidative stress in the same arterial samples. TAC increased superoxide (O_2_^-^) production in postmenopausal arteries, as indicated by lucigenin-enhanced chemiluminescence (Figure 1C). However, arteries from premenopausal women were protected from the TAC-induced increase in O_2_^-^ production (Figure 1C). In addition, we evaluated H_2_O_2_ levels with the Amplex Red assay. Premenopausal arteries did not show the TAC-dependent elevation of H_2_O_2_ production that was observed in arteries from postmenopausal women (Figure 1D).

### Effects of menopausal status on chemotherapy-induced NOX4 expression, Rho-kinase activity, and eNOS phosphorylation at Thr495 in human arteries

**Figure 2:**
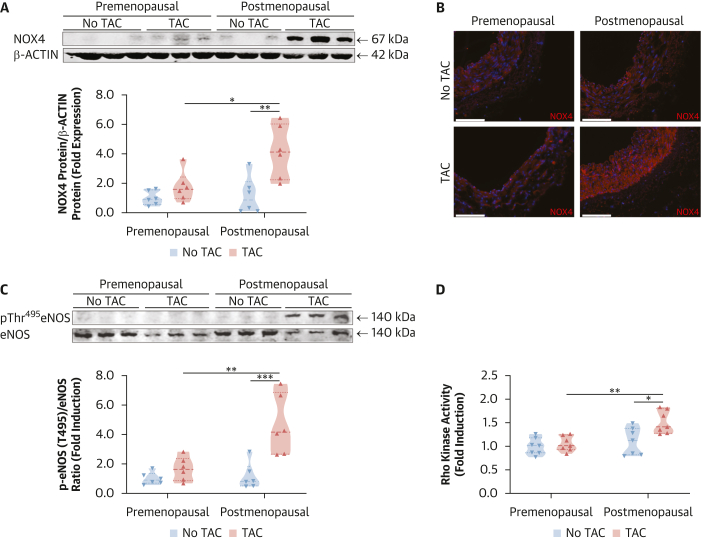
**E**ffects of Menopausal Status on Neoadjuvant Chemotherapy–Induced NOX4 Expression/Rho-Kinase Activity/p-eNOS at Thr495 Pathway (A) Protein levels of NADPH oxidase NOX4 in arteries from patients with breast cancer before and after menopause with and without prior neoadjuvant chemotherapy (TAC), with densitometric analysis normalized to β-actin (n = 6/group). Data derived from 2 independent experiments. ∗∗*P* < 0.01 vs Postmenopausal No TAC; ∗*P* < 0.05 vs Premenopausal TAC. Data were analyzed by means of 2-way ANOVA with Tukey test. Data shown as mean ± SEM. (B) Immunofluorescent identification (red) of NOX4 in vessels of the 4 patient groups. Scale bar: 150 μm. (C) Phosphorylation of eNOS at Thr495 in arteries from patients with breast cancer before and after menopause with and without prior TAC (n = 6/group) normalized to total eNOS. Data derived from 2 independent experiments. ∗∗∗*P* < 0.001 vs Postmenopausal No TAC; ∗∗*P* < 0.01 vs Premenopausal TAC. (D) Rho-kinase activity in arteries from patients with breast cancer before and after menopause with and without prior TAC (n = 7/group). ∗∗*P* < 0.01 vs Premenopausal TAC; ∗*P* < 0.05 vs Postmenopausal No TAC. (C and D) 2-way ANOVA with Tukey test. Data shown as mean ± SEM. Overall *P* values for 2-way ANOVA: (A) *P*_menopause_ = 0.0209, *P*_TAC_ = 0.0014, *P*_menopause×TAC_ = 0.0397; (C) *P*_menopause_ = 0.0044, *P*_TAC_ = 0.0004, *P*_menopause×TAC_ = 0.0091; (D) *P*_menopause_ = 0.0026, *P*_TAC_ = 0.0117, *P*_menopause×TAC_ = 0.0477.

A significant source of H_2_O_2_ in blood vessels is the NADPH oxidases, particularly NOX4. Western blotting showed that TAC did not induce NOX4 expression in arterial samples from premenopausal women, in contrast to postmenopausal patients (Figure 2A). These findings were corroborated by immunofluorescence, which also showed no detectable increase in NOX4 expression after TAC in the premenopausal group (Figure 2B). Moreover, in vitro studies in human endothelial cells showed that docetaxel induces *Nox4* mRNA expression, an effect eliminated by 17β- estradiol (Supplemental Figure 4). Nox4-derived H_2_O_2_ has been shown to induce inhibitory eNOS Thr495 phosphorylation^7^ with TAC, providing a key mechanism for TAC-induced endothelial dysfunction.

Importantly, in arteries from premenopausal women, consistent with the lack of NOX4 up-regulation, we observed no increase in eNOS phosphorylation at Thr495 in arteries from premenopausal patients treated with TAC (Figure 2C). Furthermore, we examined ROCK activity, a critical mediator of eNOS Thr495 phosphorylation. Premenopausal women showed no induction of ROCK activity after TAC, unlike their postmenopausal counterparts (Figure 2D). These findings suggest that premenopausal women are protected against docetaxel-induced vascular dysfunction by preventing NOX4 up-regulation, suppressing downstream ROCK activation, and maintaining eNOS in an active, nonphosphorylated state at Thr495.^7^^,^^22^

### Sex-dependent effects of docetaxel on endothelial function in vivo

**Figure 3:**
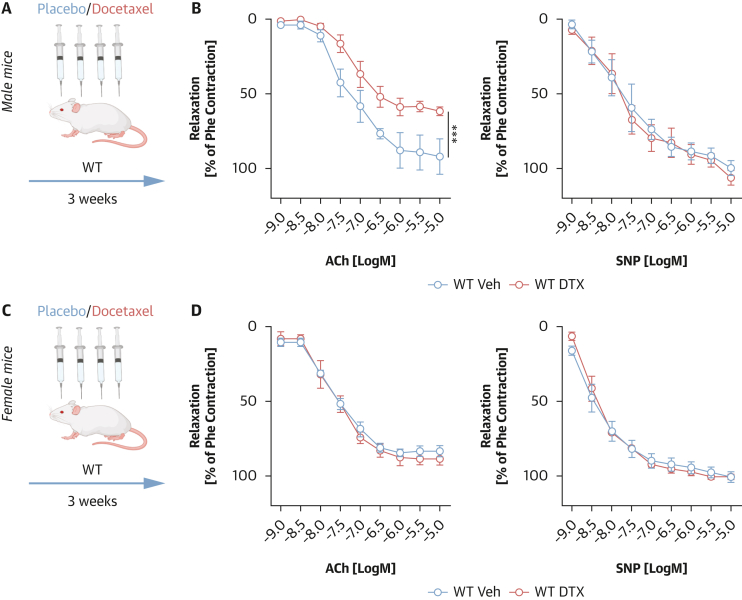
Sex-Dependent Effects of Docetaxel on Endothelial Function In Vivo (A) Study design of wild-type (WT) male mice treated with docetaxel (DTX) (10 mg/kg every 5 days for 3 weeks) or placebo. (B) Average endothelium-dependent vasorelaxations curves to acetylcholine (ACh; 1 nmol/L to 10 μmol/L, left) and endothelium-independent relaxations to sodium nitroprusside (SNP; 1 nmol/L to 10 μmol/L, right) in mouse aortas from WT male mice treated with DTX or placebo (Veh; n = 5/group). Data are expressed as mean ± SEM and analyzed by means of repeated-measures ANOVA; ∗∗∗*P* < 0.05 vs WT Veh. (C) Study design of WT female mice treated with DTX (10 mg/kg every 5 days for 3 weeks) or placebo. (D) Average endothelium-dependent vasorelaxations curves to ACh (1 nM-to 10 μmol/L, left) and endothelium-independent relaxations to SNP (1 nmol/L to 10 μmol/L, right) in mouse aortas from WT female mice treated with DTX or Veh (n = 5/group). Data are expressed as mean ± SEM and analyzed by means of repeated-measures ANOVA.

To investigate the sex-specific effects of docetaxel on vascular function, we treated C57BL/6J wild-type male and female mice with docetaxel (10 mg/kg, administered every 5 days over a 3-week period) (Figures 3A and 3C). This also enabled us to indirectly assess whether the observed protection from docetaxel-induced vascular toxicity was age or hormone dependent. In male mice, as previously described,^7^ docetaxel significantly impaired endothelium-dependent vasorelaxation in response to ACh, and endothelium-independent vasodilation to SNP remained unaffected (Figure 3B). In contrast, age-matched female mice did not exhibit any vascular dysfunction following docetaxel treatment. Both endothelium-dependent (ACh-induced) and endothelium-independent (SNP-induced) vasodilatory responses remained intact in age-matched female mice (Figure 3D), suggesting that the protective effects observed in premenopausal women extend to the in vivo female murine model.

### Ovariectomy abrogates estrogen-mediated protection against docetaxel-induced hypertension and vascular dysfunction in mice

**Figure 4:**
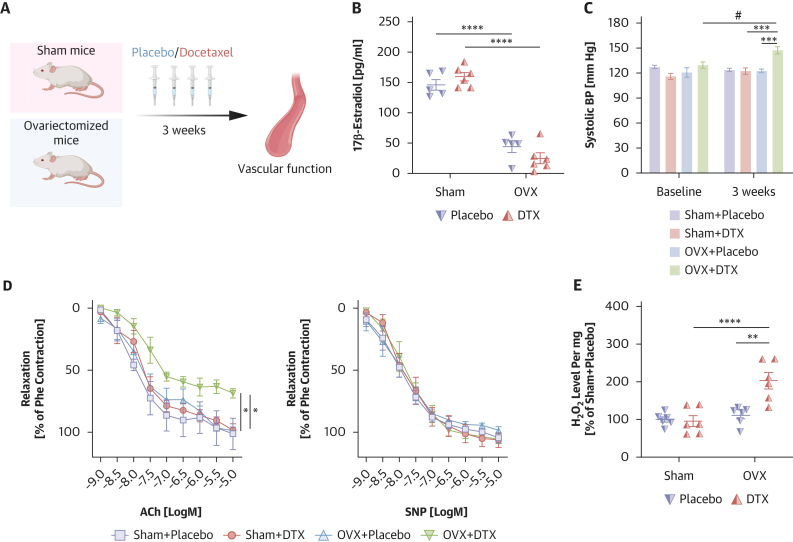
Functional Role of Estrogen in the Regulation of Vascular Dysfunction Induced by Docetaxel In Vivo (A) Study design of wild-type (WT) female mice treated with docetaxel (DTX; 10 mg/kg every 5 days for 3 weeks) or placebo with or without bilateral ovariectomy (OVX). (B) The level of 17β-estradiol in mouse plasma from WT female mice treated with DTX or placebo with or without bilateral OVX (n = 5-6/group). Data are expressed as mean ± SEM and analyzed by means of 2-way ANOVA with Tukey test; ∗∗∗∗*P* < 0.001 vs Sham+Placebo, Sham+DTX. (C) Systolic blood pressure (BP) by tail-cuff plethysmography in WT female mice treated with DTX or placebo with or without bilateral OVX (n = 5-6/group). Data are expressed as mean ± SEM and analyzed by means of 2-way repeated-measures ANOVA with Sidak test; ∗∗∗*P* < 0.001 vs Sham+DTX, OVX+Placebo; #*P* < 0.01 vs Baseline. (D) Average endothelium-dependent vasorelaxations curves to acetylcholine (ACh; 1 nmol/L to 10 μmol/L, left) and endothelium-independent relaxations to sodium nitroprusside (SNP; 1 nmol/L to 10 μmol/L, right) in mouse aortas from WT female mice treated with DTX or placebo with or without bilateral OVX (n = 5-6/group). Data are expressed as mean ± SEM and analyzed by means of 2-way repeated-measures ANOVA with Tukey test; ∗*P* < 0.05 vs Sham+DTX, OVX+Placebo. (E) Hydrogen peroxide (H_2_O_2_) production according to Amplex Red in aortas from WT female mice treated with DTX or placebo with or without bilateral OVX (n = 6/group). Data are expressed as mean ± SEM and analyzed by means of 2-way ANOVA with Tukey test; ∗∗∗*P* < 0.001 vs Sham+DTX; ∗∗*P* < 0.01 vs OVX+Placebo. Overall *P* values for 2-way ANOVA: (B) *P*_OVX_ < 0.0001, *P*_DTX_ = 0.7316, *P*_OVX×DTX_ = 0.0713; for repeated-measures 2-way ANOVA: (C) *P*_time_ = 0.0251, *P*_group_ = 0.0007, *P*_time×group_ = 0.0291; (D) for ACh: *P*_response_ < 0.0001, *P*_group_ = 0.0091, *P*_response×group_ = 0.4762; for SNP: *P*_response_ < 0.0001, *P*_group_ = 0.9916, *P*_response×group_ = 0.6355; for 2-way ANOVA: (E) *P*_OVX_ = 0.0005, *P*_DTX_ = 0.0060, *P*_OVX×DTX_ = 0.0030.

To directly assess the role of estrogen in protecting against docetaxel-induced endothelial dysfunction, independently from age and other confounders, we performed bilateral ovariectomy in female C57BL/6J mice to eliminate endogenous estrogen signaling (Figure 4A). The effectiveness of ovariectomy was confirmed by measuring circulating 17β-estradiol levels with ELISA, which showed a significant reduction compared to sham-operated control animals (Figure 4B).

Blood pressure measurements revealed that docetaxel treatment induced hypertension in ovariectomized mice, whereas no such effect was observed in sham-operated animals (Figure 4C). In line with our observations in human vascular samples, docetaxel impaired endothelium-dependent vasodilation (ACh-induced) in ovariectomized mice, whereas vascular function remained intact in age-matched nonovariectomized mice (Figure 4D). Endothelium-independent vasodilation in response to SNP was unaffected by docetaxel treatment in both groups (Figure 4D), confirming that the vascular dysfunction was endothelium specific. Consistent with our findings in human arteries (Figure 1D), docetaxel significantly increased vascular H_2_O_2_ production in ovariectomized mice, as measured by means of Amplex Red. Such oxidative stress response was absent in sham-operated female mice (Figure 4E), indicating that estrogen suppresses docetaxel-induced ROS generation.

**Figure 5:**
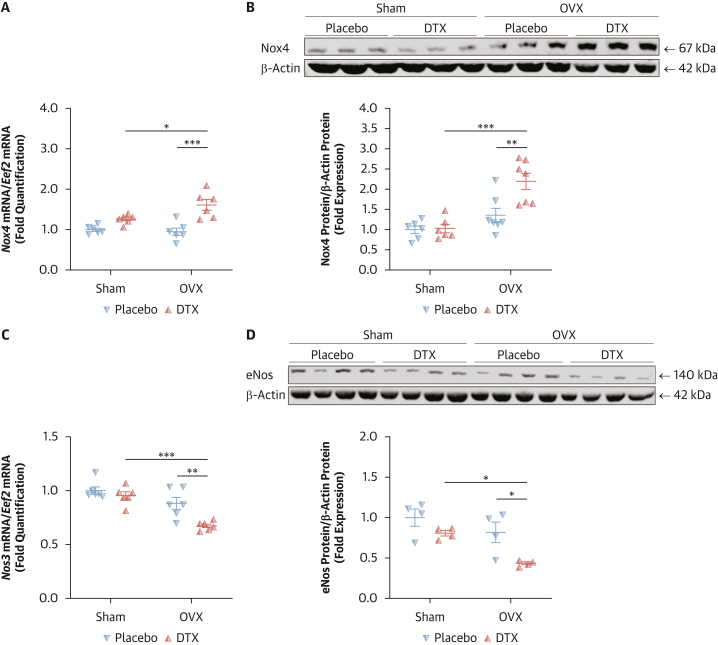
Effects of Estrogen in the Regulation of Mechanisms of Vascular Biology and Oxidative Stress After Docetaxel Treatment In Vivo (A) The level of *Nox4* mRNA expression in mouse aortas from wild-type (WT) female mice treated with docetaxel (DTX) or placebo with or without bilateral ovariectomy (OVX; n = 6/group). ∗∗∗*P* < 0.001 vs OVX+Placebo; ∗*P* < 0.05 vs Sham+DTX. (B) The level of Nox4 protein expression in mouse aortas from WT female mice treated with DTX or placebo with or without bilateral OVX (n = 6-7/group). Densitometric analysis normalized to total β-actin (top). Data derived from 2 independent experiments. ∗∗∗*P* < 0.001 vs Sham+DTX; ∗∗*P* < 0.01 vs OVX+Placebo. (C) The level of *Nos3* mRNA expression in mouse aortas from WT female mice treated with DTX or placebo with or without bilateral OVX (n = 6/group). ∗∗∗*P* < 0.001 vs Sham+DTX; ∗∗*P* < 0.01 vs OVX+Placebo. (D) The level of eNos protein expression in mouse aortas from WT female mice treated with DTX or placebo with or without bilateral OVX (n = 4/group). Densitometric analysis normalized to total β-actin (top). Data derived from 2 independent experiments. ∗*P* < 0.05 vs Sham+DTX, OVX+Placebo. Data analysis: 2-way ANOVA with Tukey test. Data shown as mean ± SEM. Overall *P* values for 2-way ANOVA: (A) *P*_OVX_ = 0.0964, *P*_DTX_ < 0.0001, *P*_OVX×DTX_ = 0.0300; (B) *P*_OVX_ < 0.0001, *P*_DTX_ = 0.0116, *P*_OVX×DTX_ = 0.0186; (C) *P*_OVX_ < 0.0001, *P*_DTX_ = 0.0029, *P*_OVX×DTX_ = 0.0422; (D) *P*_OVX_ = 0.0068, *P*_DTX_ = 0.0055, *P*_OVX×DTX_ = 0.2893.

To explore underlying mechanisms, we assessed NOX4 expression. Both qRT-PCR and Western blotting demonstrated that docetaxel increased NOX4 mRNA and protein expression in arteries from ovariectomized mice (Figures 5A and 5B). Moreover, we hypothesized that reduced NO bioavailability could also result from impaired eNOS expression. Interestingly, docetaxel treatment led to decreased eNOS mRNA and protein levels in ovariectomized mice (Figures 5C and 5D), providing a mechanistic link between oxidative stress, endothelial dysfunction, and hypertension in the absence of estrogen.

## Discussion

CVD is the leading cause of death among women with breast cancer,^23^^,^^24^ and this risk is closely associated with the cardiotoxicity of chemotherapeutic agents used in treatment.^25^, ^26^, ^27^ Notably, in older postmenopausal women, breast cancer survivors have a higher risk of dying from CVD compared with age-matched women without a history of breast cancer.^24^ Several studies have shown that in this population, noncancer causes of death—particularly CVD—surpass breast cancer itself as the most common cause of mortality.^28^^,^^29^ In postmenopausal breast cancer patients, neoadjuvant chemotherapy, particularly its component docetaxel, impairs vascular function by increasing ROS production and enhancing phosphorylation of eNOS at Thr495. This modification reduces NO bioavailability, thereby contributing to endothelial dysfunction.^7^

We demonstrate a significant NO-dependent preservation of arterial function in premenopausal women despite exposure to TAC, in contrast to postmenopausal subjects. Mechanistically, vessels from premenopausal women showed reduced ROS production, no up-regulation of NOX4, suppression of Rho kinase activity, and inhibition of eNOS phosphorylation at Thr495. Importantly, the study excluded patients who had received hormone therapy before surgery, allowing for an unconfounded assessment of potential endogenous estrogen effects. Because causal inference in human studies is limited and subject to confounding, we performed complementary in vivo experiments in mouse models of docetaxel-induced endothelial dysfunction. Docetaxel impaired endothelial function in male but not in intact age-matched female mice. In contrast, female mice subjected to bilateral ovariectomy, an established model of menopause-like physiology, developed marked endothelial dysfunction in response to docetaxel. Ovariectomy also sensitized female mice to docetaxel-induced hypertension, increased vascular ROS production, and elevated Nox4 expression, supporting a protective role of ovarian hormones in mitigating chemotherapy-induced vascular injury. Importantly, these effects were independent from age. The clinical relevance of this study is strong, because we used a well established model^7^ with human arterial segments obtained from women previously treated with TAC chemotherapy. Access to vessels collected during mastectomy allowed direct ex vivo assessment of vascular function, providing unique mechanistic insights into human vascular biology. Lack of impairment of vascular function by TAC in premenopausal patients may explain lower age-adjusted long-term cardiovascular risk in premenopausal compared with postmenopausal cancer survivors.^24^

Menopausal status reflects the transition from reproductive to nonreproductive life and is defined biologically by declining ovarian estrogen production and clinically by the permanent cessation of menses, and typically occurs around the age of 50 years, encompassing both chronologic aging and hormone-dependent vascular and metabolic changes. Although the precise contribution of estrogen to the vascular responses to TAC in premenopausal and postmenopausal women is difficult to determine owing to multiple confounders, including primarily age,^30^ estrogen is well established as a protective factor against CVD.^31^^,^^32^ To address the influence of estrogen, we used a widely used mouse model of menopause^33^ involving bilateral ovariectomy, which is well established in cardiovascular research.^32^^,^^34^^,^^35^ Estrogen deficiency in mice leads to increased production of vascular ROS and an enhancement of angiotensin II–induced vasoconstriction through increased expression of the vascular angiotensin II receptor type 1, leading to endothelial dysfunction.^12^

Estrogens, acting through estrogen receptors (ERs) α and β, stimulate eNOS activation and NO production, an effect blocked by N^G^-nitro-L-arginine methyl ester.^36^ This occurs via both nongenomic and genomic pathways.^37^^,^^38^ Nongenomic mechanisms involve PI3K/Akt signaling from membrane- or cytoplasm-associated ERs, leading to eNOS activation, dissociation of eNOS from caveolin, and enhanced cofactor tetrahydrobiopterin availability.^39^^,^^40^ Genomic pathways involve nuclear ER-mediated up-regulation of eNOS gene transcription, increasing eNOS protein expression and NO generation. These mechanisms are receptor isoform and tissue specific, reflecting the complexity of estrogen-mediated NO production.^41^ Interestingly, we also found that docetaxel increased blood pressure in ovariectomized female mice. Notably, ovariectomy has previously been shown to potentiate the classical hypertensive responses to angiotensin II.^32^

A combination of human vessel studies and experimental models provides insight into the potential mechanisms underlying the protection from TAC-induced vascular dysfunction observed in premenopausal women. These findings build on our previous work, which identified a novel docetaxel-driven mechanism of vascular dysfunction in vessels from postmenopausal patients. Importantly, the dysfunction is dependent on Nox4-induced H_2_O_2_ and Nox2-dependent superoxide overproduction.^7^ The causality of Nox4 in this relationship has been demonstrated by *Nox4*^−/−^ mice, which are protected from development of docetaxel-induced vascular pathology and docetaxel-induced pathology in male mice.^7^ In that model, we demonstrated that NOX4-derived H_2_O_2_ stimulates ROCK expression and activation.

ROCK is a key kinase mediating inhibitory phosphorylation of eNOS in endothelial cells,^22^ and our previous work established its mechanistic role in TAC-induced vascular dysfunction.^7^ Moreover, docetaxel directly increased ROCK activity in human dermal microvascular endothelial cells. Enhanced phosphorylation of eNOS on Thr 495 caused by docetaxel in vitro and ex vivo was also significantly reduced by the Rho kinase inhibitor. Faraco et al^42^ also showed that the ROCK inhibitor prevented the interleukin-17–induced eNOS phosphorylation and attenuated ACh-induced NO increase. In mice, administration of fasudil, a ROCK inhibitor, prevented docetaxel-induced increases in blood pressure and vascular dysfunction.^7^ That ROCK inhibitor reduced docetaxel-induced Thr495 eNOS phosphorylation, further supporting the role of ROCK in docetaxel-induced eNOS dysfunction. Notably, those changes occurred in wild-type but not *Nox4*^−/−^ mice, supporting a NOX4-ROCK-eNOS pathway in docetaxel vascular toxicity.^7^

We have now extended understanding of these mechanisms by showing that premenopausal status in women and female sex in mice confer protection against TAC- and docetaxel-induced NOX4 overexpression and affect all elements of the Nox4–ROCK–eNOS–Thr495 phosphorylation–NO pathway. These findings align with mechanistic studies showing that estrogen inhibits ROCK activity in the cerebral circulation and reduces ROCK expression in cultured human coronary artery vascular smooth muscle cells.^43^^,^^44^ However, further studies are needed. Estrogen replacement in ovariectomized mice could clarify whether estrogen prevents docetaxel-induced endothelial dysfunction in this setting. In addition, assessing vascular function in ovariectomized mice treated with a Nox4 inhibitor would be informative in the context of menopausal status, building on robust evidence from postmenopausal human vessels and mouse models.^7^

Future studies are therefore needed for definitive evidence, but in human endothelial cell culture we observed a clear inhibitory effect of estrogen on NOX4 expression, providing proof of concept for a causal interaction. This supports further exploration of therapeutic strategies to mitigate docetaxel vascular toxicity. While estrogen supplementation is not an option in this patient group, delineating the signaling pathways connecting estrogen to NOX4 biology may reveal alternative therapeutic targets. Interestingly, in ovariectomized mice in addition to eNOS Thr495 phosphorylation, decreased bioavailability of NO after docetaxel treatment could also be attributed to the diminished expression of eNOS.

### Study Limitations

Several limitations of this study warrant careful consideration. First, the cross-sectional design and modest sample size preclude definitive causal inference. Although we matched groups for cardiovascular risk factors, premenopausal women were significantly younger than postmenopausal women, as expected,^30^ and we cannot completely exclude an independent effect of age on vascular function despite the mechanistic data supporting a hormonal mechanisms. To address the influence of age, we performed experiments in age-matched male and female animals and added age-adjusted statistical analyses in patients. However, given the collinearity of age and menopause and the magnitude of the age difference in patients, an independent effect of age cannot be fully excluded, particularly as aging itself is accompanied by major hormonal shifts. At the same time, it is important to note that longitudinal cohort studies (eg, SWAN) show menopause-related endothelial decline extends beyond sole chronologic aging, and later age at menopause is associated with better endothelial function,^14^ concordantly with our interpretation. Second, patients were receiving a multiagent TAC regimen; although our previous findings implicate docetaxel as the principal mediator of endothelial toxicity,^6^ concomitant exposure to doxorubicin and cyclophosphamide may contribute to the observed vascular effects in patients. Third, although differences in cardiovascular medication use (eg, beta-blockers, angiotensin-converting enzyme inhibitors) did not reach statistical significance, such treatments may influence endothelial function and oxidative stress. We therefore performed additional analyses excluding postmenopausal patients on these medications, and all findings were maintained. Fourth, we focused on women who had not yet received ovarian function suppression or endocrine therapy; therefore, our results may be most applicable to premenopausal women treated with TAC chemotherapy alone. Notwithstanding these limitations, our findings generate hypotheses about hormone-dependent vascular protection that merit further exploration and have potential implications for cardio-oncology risk stratification.

## Conclusions

This study defines a practical framework for vascular cardio-oncology: docetaxel activates a NOX4-ROCK-eNOS axis, and its engagement is modified by menopausal status. Clinically, this supports using menopausal or hormonal status as a risk modifier in docetaxel-containing cancer therapies, with pragmatic steps that can be implemented now: baseline vascular profiling (blood pressure, lipids, glycemia, smoking, body mass index), medication review, and early cardio-oncology input in postmenopausal or multimorbid patients. On-treatment surveillance should focus on blood pressure trends, volume status, and pleural or pericardial effusions each cycle, as well as targeted follow-up in survivorship clinics prioritizing postmenopausal women and those with cardiometabolic risk. In the future, noninvasive vascular assessments (eg, endothelial function or arterial stiffness measures) may refine risk, but routine hemodynamic monitoring remains the core. Our data also provide a mechanistic rationale for considering mechanism-aligned prevention and interventions in high-risk patients. Inhibitors of NOX1/4 (eg, setanaxib, which is currently available in human trials) and ROCK (fasudil) have shown efficacy in preclinical models.^7^ Moreover, statins and angiotensin-converting enzyme inhibitors/angiotensin receptor blockers with nebivolol when appropriate may offer clinically available protection by suppressing ROS and improving NO-cGMP signaling, while controlling blood pressure. Importantly, strategies should be tailored to estrogen status, with postmenopausal or aromatase inhibitor–treated women prioritized for NOX/ROCK–targeted therapies and exploratory trials of eNOS recoupling agents such as tetrahydrobiopterin (eg, sapropterin). Importantly, such selective inhibition of the NOX4-ROCK-eNOS axis can likely preserve vascular function without compromising the antitumor efficacy of docetaxel.

To our knowledge, this work offers the first mechanistic explanation for the selectively increased cardiovascular risk observed in postmenopausal breast cancer survivors,^45^ with potential to translate biology into immediate implementable clinical vigilance. Our study addresses this gap by linking docetaxel exposure to a defined endothelial pathway and showing that engagement of this pathway differs by menopausal status, thereby generating testable hypotheses for risk stratification (eg, hormonal milieu), surveillance (eg, blood pressure and effusion monitoring, endothelial function metrics),^46^ and mechanism-based prevention.^47^ Beyond the scope of the present study, women with ER-positive breast cancer receiving ovarian suppression plus aromatase inhibitor therapy enter an iatrogenic estrogen-deficient state similar to menopause, which may amplify susceptibility to docetaxel-induced oxidative stress and vascular dysfunction and warrants dedicated evaluation in that high-risk subgroup.

**Figure 6:**
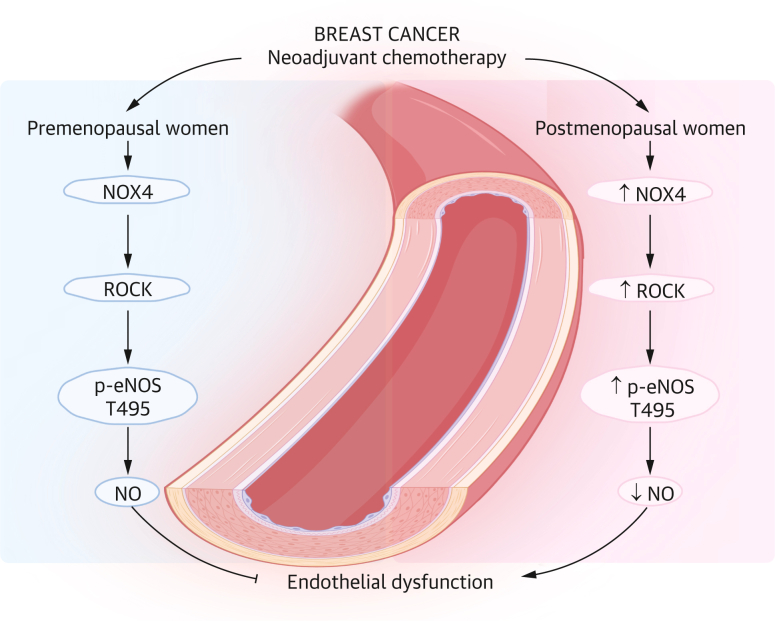
Mechanism of Neoadjuvant Chemotherapy on Vascular Function in Breast Cancer Patients Neoadjuvant chemotherapy induces endothelial dysfunction in postmenopausal women via the NOX4–ROCK–p-eNOS pathway. Premenopausal women are protected from neoadjuvant chemotherapy–induced endothelial dysfunction. eNOS = endothelial nitric oxide synthase; NO = nitric oxide; ROCK = rho-associated protein kinase.

In conclusion, the present study demonstrates that premenopausal status and estrogen signaling confer protection against endothelial dysfunction and oxidative stress induced by neoadjuvant chemotherapy (TAC), particularly docetaxel, in postmenopausal women and male mice (Figure 6, Central Illustration). Estrogens help preserve endothelial NO production, thereby maintaining vascular homeostasis and preventing the decline in endothelial function that can increase the risk of vascular disease. These findings uncover a previously unrecognized vascular effect of docetaxel and highlight a novel potentially estrogen-dependent mechanism by which premenopausal patients are protected from chemotherapy-induced vascular injury in breast cancer. Most importantly, our findings underscore the need for increased focus on primary prevention in breast cancer survivors, particularly in postmenopausal women.

**Central Illustration:**
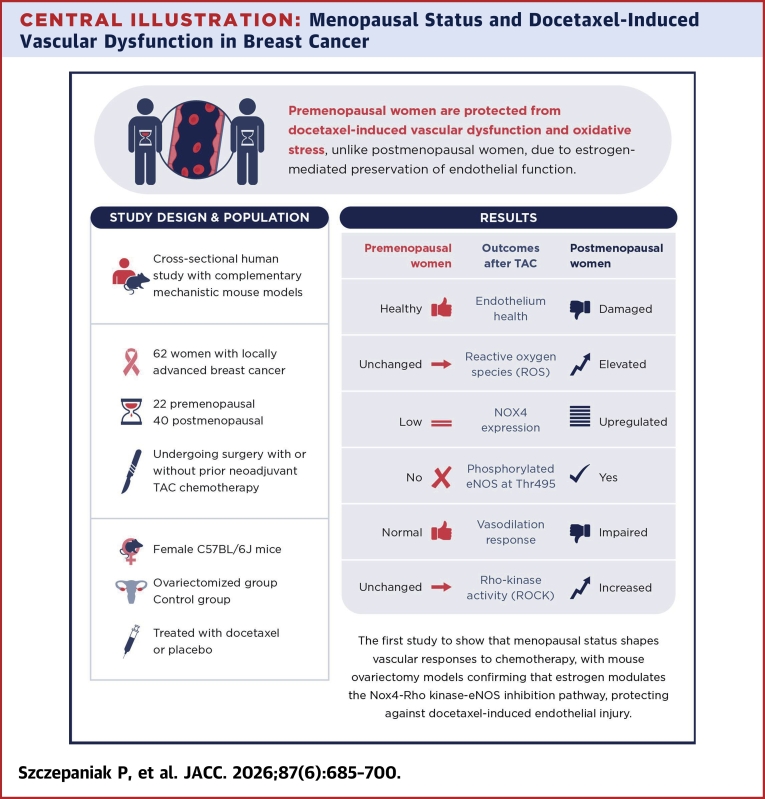
Menopausal Status and Docetaxel-Induced Vascular Dysfunction in Breast Cancer eNOS = endothelial nitric oxide synthase; TAC = neoadjuvant chemotherapy (cyclophosphamide-docetaxel-doxorubicin).

### Data Availability

Supporting data are available from the corresponding author upon reasonable request.

## Funding Support and Author Disclosures

This study was funded by the National Science Center, Poland (2023/51/D/NZ4/00270 to P.S.), British Heart Foundation (PG/22/11041 and SP/F/24/150068 to T.J.G.), and European Commission–NCBiR, Poland (ERA-CVD/Gut-brain/8/2021 and ImmuneHyperCog to T.J.G.). The authors have reported that they have no relationships relevant to the contents of this paper to disclose.

## References

[1] Harbeck N., Gnant M. (2017). Breast cancer. Lancet.

[2] Kirkham A.A., Beaudry R.I., Paterson D.I., Mackey J.R., Haykowsky M.J. (2019). Curing breast cancer and killing the heart: a novel model to explain elevated cardiovascular disease and mortality risk among women with early stage breast cancer. Prog Cardiovasc Dis.

[3] Hooning M.J., Botma A., Aleman B.M. (2007). Long-term risk of cardiovascular disease in 10-year survivors of breast cancer. J Natl Cancer Inst.

[4] Strongman H., Gadd S., Matthews A. (2019). Medium and long-term risks of specific cardiovascular diseases in survivors of 20 adult cancers: a population-based cohort study using multiple linked UK electronic health records databases. Lancet.

[5] Peclat T.R., Agorrody G., Colman L. (2024). Ecto-CD38-NADase inhibition modulates cardiac metabolism and protects mice against doxorubicin-induced cardiotoxicity. Cardiovasc Res.

[6] Canonico F., Pedicino D., Severino A. (2023). GLUT-1/PKM2 loop dysregulation in patients with non-ST-segment elevation myocardial infarction promotes metainflammation. Cardiovasc Res.

[7] Szczepaniak P., Siedlinski M., Hodorowicz-Zaniewska D. (2022). Breast cancer chemotherapy induces vascular dysfunction and hypertension through a NOX4-dependent mechanism. J Clin Invest.

[8] Virani S.S., Alonso A., Benjamin E.J. (2020). Heart disease and stroke statistics—2020 update: a report from the American Heart Association. Circulation.

[9] SenthilKumar G., Katunaric B., Bordas-Murphy H., Sarvaideo J., Freed J.K. (2023). Estrogen and the vascular endothelium: the unanswered questions. Endocrinology.

[10] Iorga A., Cunningham C.M., Moazeni S., Ruffenach G., Umar S., Eghbali M. (2017). The protective role of estrogen and estrogen receptors in cardiovascular disease and the controversial use of estrogen therapy. Biol Sex Differ.

[11] Lu Q., Schnitzler G.R., Ueda K. (2016). ER alpha rapid signaling is required for estrogen induced proliferation and migration of vascular endothelial cells. PLoS One.

[12] Wassmann S., Bäumer A.T., Strehlow K. (2001). Endothelial dysfunction and oxidative stress during estrogen deficiency in spontaneously hypertensive rats. Circulation.

[13] Green D.J., Dawson E.A., Groenewoud H.M., Jones H., Thijssen D.H. (2014). Is flow-mediated dilation nitric oxide mediated? A meta-analysis. Hypertension.

[14] Moreau K.L., Hildreth K.L., Meditz A.L., Deane K.D., Kohrt W.M. (2012). Endothelial function is impaired across the stages of the menopause transition in healthy women. J Clin Endocrinol Metab.

[15] Patnaik J.L., Byers T., DiGuiseppi C., Dabelea D., Denberg T.D. (2011). Cardiovascular disease competes with breast cancer as the leading cause of death for older females diagnosed with breast cancer: a retrospective cohort study. Breast Cancer Res.

[16] Surakasula A., Nagarjunapu G.C., Raghavaiah K.V. (2014). A comparative study of pre- and post-menopausal breast cancer: risk factors, presentation, characteristics and management. J Res Pharm Pract.

[17] Partridge A.H., Hughes M.E., Ottesen R.A. (2012). The effect of age on delay in diagnosis and stage of breast cancer. Oncologist.

[18] Idris A.I. (2012). Ovariectomy/orchidectomy in rodents. Methods Mol Biol.

[19] Khajuria D.K., Razdan R., Mahapatra D.R. (2012). Description of a new method of ovariectomy in female rats. Rev Bras Reumatol.

[20] Nosalski R., Siedlinski M., Denby L. (2020). T-Cell–derived miRNA-214 mediates perivascular fibrosis in hypertension. Circ Res.

[21] Guzik T.J., West N.E., Black E. (2000). Vascular superoxide production by NAD(P)H oxidase: association with endothelial dysfunction and clinical risk factors. Circ Res.

[22] Sugimoto M., Nakayama M., Goto T.M., Amano M., Komori K., Kaibuchi K. (2007). Rho-kinase phosphorylates eNOS at threonine 495 in endothelial cells. Biochem Biophys Res Commun.

[23] Mehta L.S., Watson K.E., Barac A. (2018). Cardiovascular disease and breast cancer: where these entities intersect: a scientific statement from the American Heart Association. Circulation.

[24] Bradshaw P.T., Stevens J., Khankari N., Teitelbaum S.L., Neugut A.I., Gammon M.D. (2016). Cardiovascular disease mortality among breast cancer survivors. Epidemiology.

[25] McLaughlin M., Florida-James G., Ross M. (2021). Breast cancer chemotherapy vascular toxicity: a review of mediating mechanisms and exercise as a potential therapeutic. Vasc Biol.

[26] Chen J., Wei J., Xia P. (2024). Inhibition of cyclin-dependent kinase 7 mitigates doxorubicin cardiotoxicity and enhances anticancer efficacy. Cardiovasc Res.

[27] Lee S.H., Lee J., Oh J. (2024). Inhibition of TBL1 cleavage alleviates doxorubicin-induced cardiomyocytes death by regulating the Wnt/β-catenin signal pathway. Cardiovasc Res.

[28] Chapman J.A., Meng D., Shepherd L. (2008). Competing causes of death from a randomized trial of extended adjuvant endocrine therapy for breast cancer. J Natl Cancer Inst.

[29] Hanrahan E.O., Gonzalez-Angulo A.M., Giordano S.H. (2007). Overall survival and cause-specific mortality of patients with stage T1a,bN0M0 breast carcinoma. J Clin Oncol.

[30] Peacock K., Carlson K., Ketvertis K.M. (December 21, 2023). Menopause. StatPearls.

[31] Liu S.L., Bajpai A., Hawthorne E.A. (2019). Cardiovascular protection in females linked to estrogen-dependent inhibition of arterial stiffening and macrophage MMP12. JCI Insight.

[32] Dutta S.R., Singh P., Malik K.U. (2023). Ovariectomy via 12/15-lipoxygenase augments angiotensin II–induced hypertension and its pathogenesis in female mice. Hypertension.

[33] Diaz Brinton R. (2012). Minireview: translational animal models of human menopause: challenges and emerging opportunities. Endocrinology.

[34] Kilanowski-Doroh I.M., McNally A.B., Wong T.J. (2024). Ovariectomy-induced arterial stiffening differs from vascular aging and is reversed by GPER activation. Hypertension.

[35] Dinh Q.N., Vinh A., Kim H.A. (2021). Aldosterone-induced hypertension is sex-dependent, mediated by T cells and sensitive to GPER activation. Cardiovasc Res.

[36] Townsend E.A., Meuchel L.W., Thompson M.A., Pabelick C.M., Prakash Y.S. (2011). Estrogen increases nitric-oxide production in human bronchial epithelium. J Pharmacol Exp Ther.

[37] Xiang D., Liu Y., Zhou S., Zhou E., Wang Y. (2021). Protective effects of estrogen on cardiovascular disease mediated by oxidative stress. Oxid Med Cell Longev.

[38] Kauser K., Rubanyi G.M. (1997). Potential cellular signaling mechanisms mediating upregulation of endothelial nitric oxide production by estrogen. J Vasc Res.

[39] Feron O., Saldana F., Michel J.B., Michel T. (1998). The endothelial nitric-oxide synthase-caveolin regulatory cycle. J Biol Chem.

[40] Moreau K.L., Meditz A., Deane K.D., Kohrt W.M. (2012). Tetrahydrobiopterin improves endothelial function and decreases arterial stiffness in estrogen-deficient postmenopausal women. Am J Physiol Heart Circ Physiol.

[41] Liu L., Wang Z. (2013). Estrogen attenuates lipopolysaccharide-induced nitric oxide production in macrophages partially via the nongenomic pathway. Cell Immunol.

[42] Faraco G., Brea D., Garcia-Bonilla L. (2018). Dietary salt promotes neurovascular and cognitive dysfunction through a gut-initiated T_H_17 response. Nat Neurosci.

[43] Chrissobolis S., Budzyn K., Marley P.D., Sobey C.G. (2004). Evidence that estrogen suppresses rho-kinase function in the cerebral circulation in vivo. Stroke.

[44] Hiroki J., Shimokawa H., Mukai Y., Ichiki T., Takeshita A. (2005). Divergent effects of estrogen and nicotine on rho-kinase expression in human coronary vascular smooth muscle cells. Biochem Biophys Res Commun.

[45] Bardia A., Arieas E.T., Zhang Z. (2012). Comparison of breast cancer recurrence risk and cardiovascular disease incidence risk among postmenopausal women with breast cancer. Breast Cancer Res Treat.

[46] Heiss C., Rodriguez-Mateos A., Bapir M., Skene S.S., Sies H., Kelm M. (2023). Flow-mediated dilation reference values for evaluation of endothelial function and cardiovascular health. Cardiovasc Res.

[47] Tonry C., Russell-Hallinan A., McCune C. (2023). Circulating biomarkers for management of cancer therapeutics-related cardiac dysfunction. Cardiovasc Res.

