# Supplementary material for: Menopausal Status Associated With Docetaxel-Induced Vascular Dysfunction in Breast Cancer Patients
Source: J Am Coll Cardiol. 2026 Feb 17;87(6):685–700. doi: 10.1016/j.jacc.2025.10.077 (PMC12900610; doi:10.1016/j.jacc.2025.10.077)
Supplement: Supplemental Material [file mmc1.docx]

**SUPPLEMENTAL MATERIAL:**

**Menopausal Status Associated With Docetaxel-Induced Vascular Dysfunction in Breast Cancer Patients**

Piotr Szczepaniak, PhD,^a^ Tomasz P. Mikolajczyk, PhD,^a^ Ewelina Jozefczuk, PhD,^a^ Diana Hodorowicz-Zaniewska, MD, PhD,^b^ Joanna Streb, MD, PhD,^c^ Jakub Jurczyk, MSc,^d^ Ryszard Nosalski, PhD,^e^ Mateusz Siedlinski, PhD,^a^ Paulina Sajdak, MD,^b^ Karolina Brzuszkiewicz, MD,^b^ Mateusz Gorski, MD,^b^ Maciej Tomaszewski, MD,^f,g^ Joanna Sulicka-Grodzicka, MD, PhD,^h^ Iwona Laksa, MD,^c^ Tomasz Grodzicki, MD, PhD,^i^ Tomasz J. Guzik, MD, PhD^a,e^

From the ^a^Department of Internal and Agricultural Medicine, Jagiellonian University Medical College, Cracow, Poland; ^b^Breast Unit, Department of Surgery, Jagiellonian University Hospital, Cracow, Poland; ^c^Department of Oncology, Jagiellonian University Medical College, Cracow, Poland; ^d^Department of Toxicological Biochemistry, Jagiellonian University Medical College, Cracow, Poland; ^e^Centre for Cardiovascular Science, Queen’s Medical Research Institute, University of Edinburgh, Edinburgh, United Kingdom; ^f^Division of Cardiovascular Sciences, Faculty of Medicine, Biology and Health, University of Manchester, Manchester, United Kingdom; ^g^Division of Medicine and Manchester Academic Health Science Centre, Manchester University NHS Foundation Trust Manchester, Manchester, United Kingdom; ^h^Department of Rheumatology, Jagiellonian University Medical College, Cracow, Poland; and the ^i^Department of Internal Medicine and Gerontology, Jagiellonian University Medical College, Cracow, Poland.

**
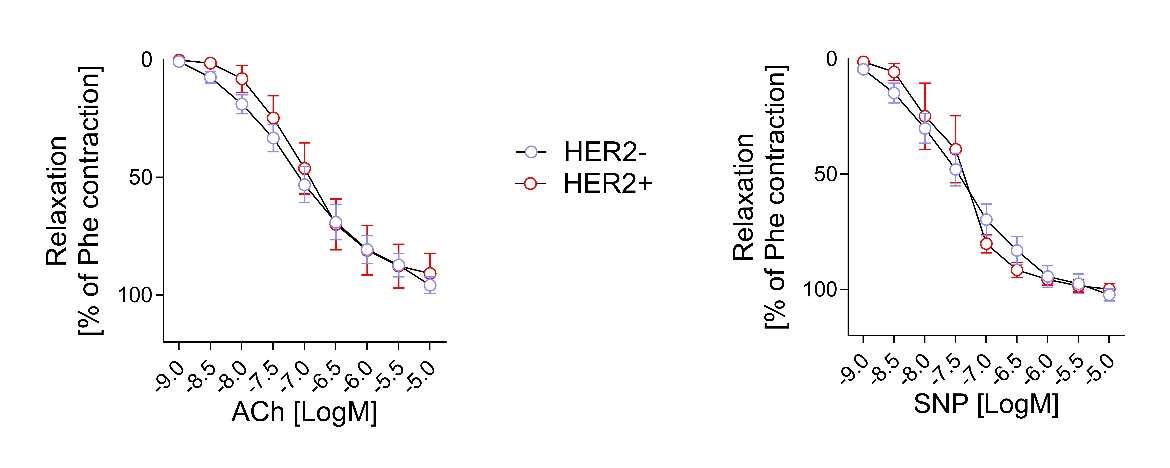
**

**Supplemental Figure 1: Effects of HER2 on vascular function in breast cancer patients**

Average endothelium-dependent vasorelaxations curves to acetylcholine (ACh; 1 nM to 10 μM, left) and endothelium-independent relaxations to sodium nitroprusside (SNP; 1 nM to 10 μM, right) in blood vessels from breast cancer patients without TAC (No TAC) with human epidermal growth factor receptor 2-negative (HER2-) and HER2-positive (HER2+). Data are expressed as mean ± s.e.m. Data were analyzed with repeated measures ANOVA.

**
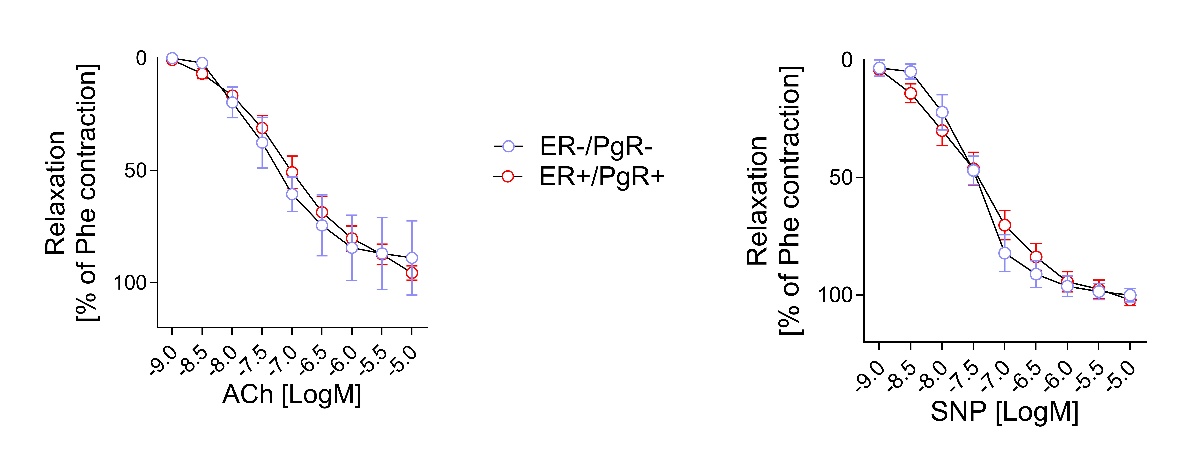
**

**Supplemental Figure 2: Effects of ER/PgR on vascular function in breast cancer patients**

Average endothelium-dependent vasorelaxations curves to acetylcholine (ACh; 1 nM to 10 μM, left) and endothelium-independent relaxations to sodium nitroprusside (SNP; 1 nM to 10 μM, right) in blood vessels from breast cancer patients without TAC (No TAC) with estrogen receptror/progesterone receptor-negative (ER-/PgR-) and ER/PgR-positive (ER+/PgR+). Data are expressed as mean ± s.e.m. Data were analyzed with repeated measures ANOVA.


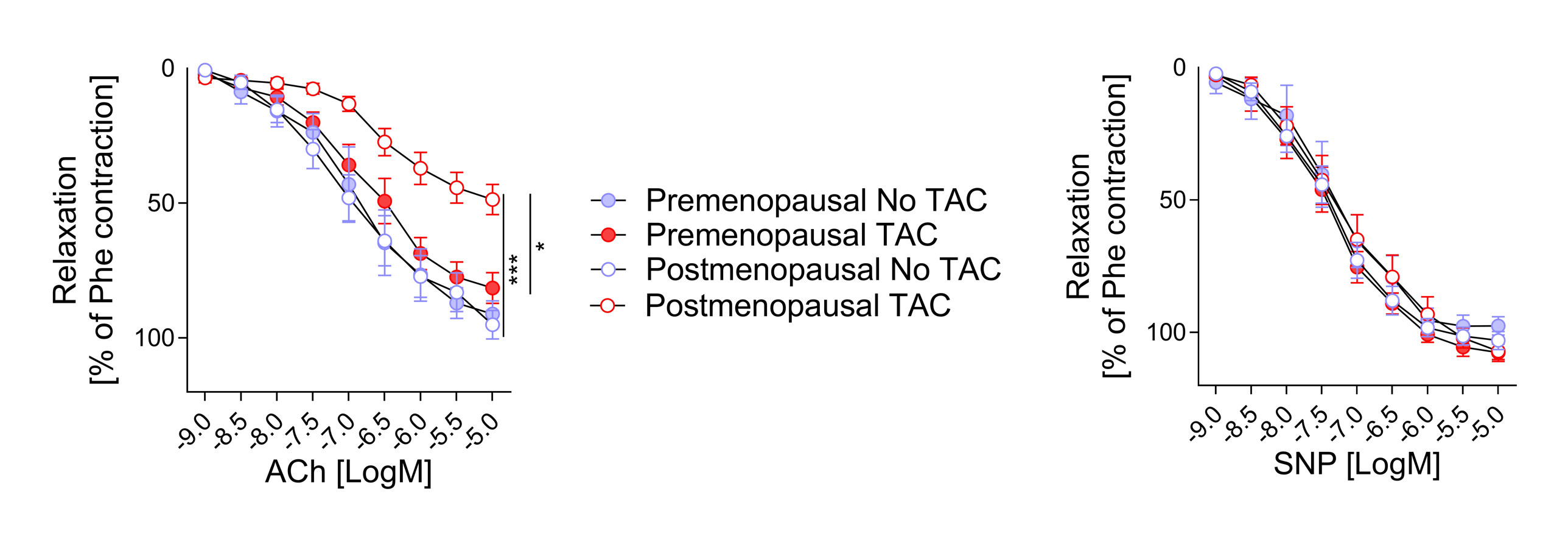


**Supplemental Figure 3: Effects of menopausal status on neoadjuvant chemotherapy (TAC)-induced endothelial dysfunction in patients without ACEI/ARB and β-blocker treatment**

Average endothelium-dependent vasorelaxations curves to acetylcholine (ACh; 1 nM to 10 μM, left) and endothelium-independent relaxations to sodium nitroprusside (SNP; 1 nM to 10 μM, right) in blood vessels from premenopausal and postmenopausal women without TAC (No TAC) and from patients who underwent TAC (n=8-15). Data are expressed as mean ± s.e.m. ***P < 0.001 vs. Postmenopausal No TAC; *P < 0.01 vs. Premenopausal TAC. Data were analyzed with two-way repeated measures ANOVA with Sidak’s test. Overall *P* values for repeated measures 2-way ANOVA for ACh: P^response^<0.0001, P^group^=0.0002, P^response x group^<0.0001; for SNP: P^response^<0.0001, P^group^=0.7484, P^response x group^=0.9976.


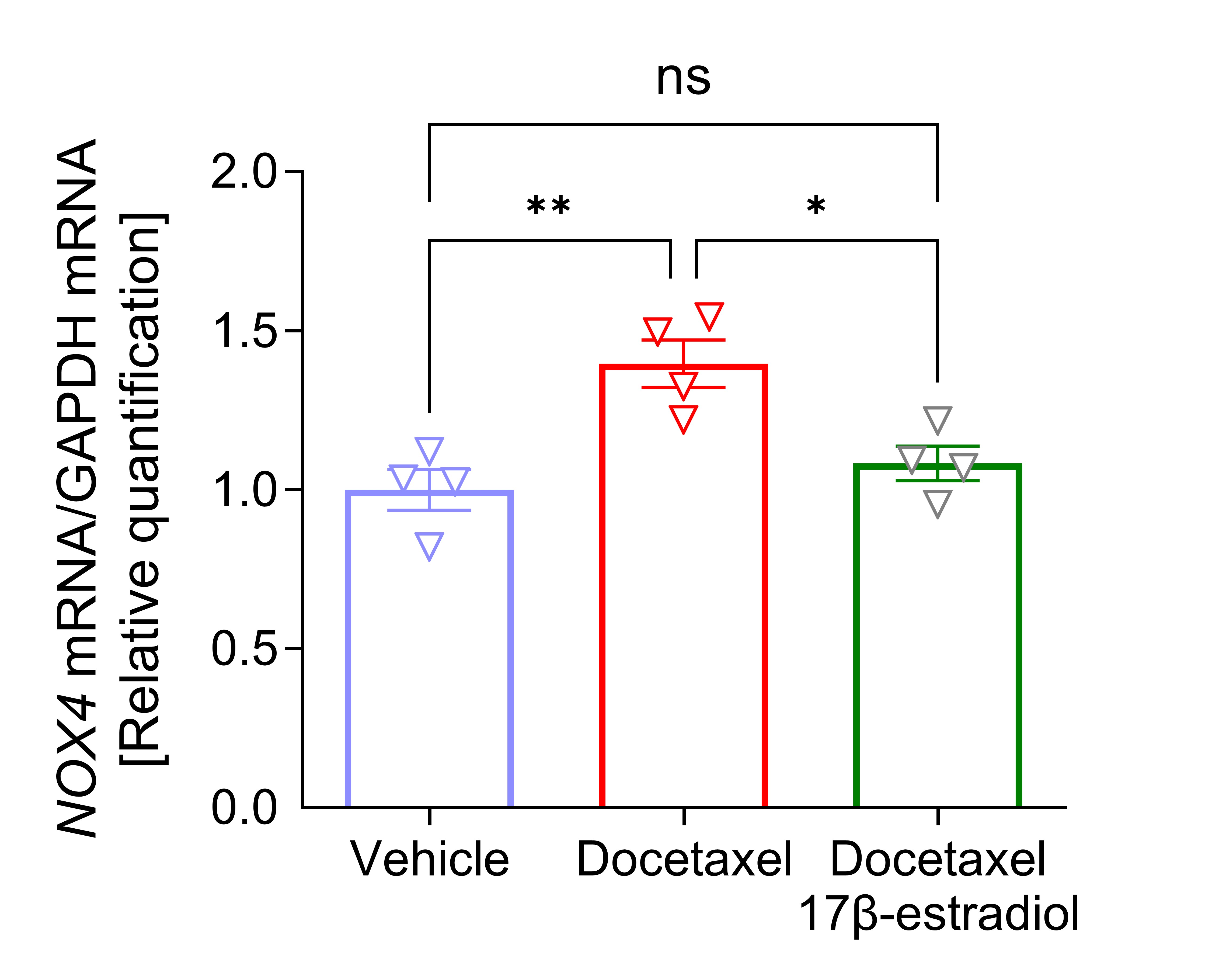


**Supplemental Figure 4: Effects of docetaxel with 17β-estradiol on *NOX4 in vitro***

Effects of 24h docetaxel (100 nM) and docetaxel (100 nM) with 17β-estradiol (100 nM) exposure on *NOX4* mRNA in Human Microvascular Endothelial Cells (HMEC-1) (n=4/group). Mean±s.e.m. **P<0.01 vs. Veh; *P<0.05 vs. Docetaxel+17β-estradiol; Data were analyzed with one-way ANOVA with Tukey’s test.

**Supplemental Table 1: Parameter estimates derived from repeated measures ANOVA comparing TAC-pretreated pre- and postmenopausal groups**

|  | No adjustment for age;  **P value** for the interaction between ACh dose and menopause status **= 0.001** | | |  | Adjusted for age;  **P value** for the interaction between ACh dose and menopause status **= 0.007** | | |
| --- | --- | --- | --- | --- | --- | --- | --- |
| ACh dose in M | Beta (Vasodilation in %, Postmenopause - Premenopause) | SE | p value |  | Beta (Vasodilation in %, Postmenopause - Premenopause) | SE | p value |
| 10^-9^ | 0.153 | 1.619 | 0.925 |  | 3.275 | 3.112 | 0.301 |
| 3x10^-9^ | -4.030 | 2.160 | 0.071 |  | 3.611 | 3.928 | 0.365 |
| 10^-8^ | -6.766 | 2.928 | 0.027 |  | 4.740 | 5.221 | 0.371 |
| 3x10^-8^ | -13.960 | 3.759 | 0.001 |  | -5.746 | 7.181 | 0.430 |
| 10^-7^ | -23.453 | 7.206 | 0.003 |  | -22.418 | 14.156 | 0.123 |
| 3x10^-7^ | -21.603 | 9.118 | 0.024 |  | -26.193 | 17.887 | 0.153 |
| 10^-6^ | -32.249 | 8.072 | 0.000 |  | -44.738 | 15.641 | 0.008 |
| 3x10^-6^ | -34.332 | 7.588 | 0.000 |  | -43.796 | 14.776 | 0.006 |
| 10^-5^ | -34.873 | 7.571 | 0.000 |  | -43.504 | 14.764 | 0.006 |

ACh, acetylcholine.
